# *MET* fusions and splicing variants in glioma: a landscape integrating clinical, pathological, and survival features

**DOI:** 10.1002/2056-4538.70085

**Published:** 2026-03-30

**Authors:** Zheng Fang, Chengjun Zheng, Peng Wang, Xing Liu, Lingyu Liu, Guanzhang Li, Jiahan Dong, Qiaodong Chen, Delong Zhang, Yutong Feng, Ying Zhang, Zhaoshi Bao

**Affiliations:** ^1^ Department of Molecular Neuropathology Beijing Neurosurgical Institute, Capital Medical University Beijing PR China; ^2^ Department of Neurosurgery Beijing Tiantan Hospital, Capital Medical University Beijing PR China; ^3^ Department of Neuropathology Beijing Tiantan Hospital, Capital Medical University Beijing PR China

**Keywords:** glioma, mesenchymal–epidermal, transition factor, MET F/SVs, integrated analysis

## Abstract

*MET* alterations, including *MET* fusions and splicing variants (F/SVs), are linked to glioma progression, but the clinical features remain underexplored since the 2021 WHO classification of tumors of the CNS. We aimed to systematically depict the *MET* F/SVs and patient characteristics in a multicenter cohort focusing on clinical, pathological, and survival features. We studied data from 1,041 patients with *MET* F/SVs data from the public Chinese Glioma Genome Atlas database and the TruSight Tumor 170 study. Clinical outcomes were evaluated based on the RANO criteria. We used chi‐square and Fisher's exact tests for variable analysis. Kaplan–Meier analysis was used to assess survival trends, while univariate and multivariate analyses revealed the prognostic value of *MET* F/SVs. Immunohistochemical staining was performed to demonstrate the MET expression level. Among the 1,041 patients, 49 patients had F/SVs (4.70%), and 23 had ZM fusion (*PTPRZ1‐MET* fusion gene; 2.21%). Among the 67 recurrent grade 4 astrocytomas, the proportions of F/SVs (11.94%, *n* = 8) and ZMs (5.97%, *n* = 4) were the highest. *MET* F/SVs were significantly associated with malignant clinical outcomes in the *IDH*‐mutant astrocytoma cohort, with a frequency of 5.04% (18/357) across all WHO grades. Multivariate analysis revealed that the *MET* F/SVs were independently associated with worse survival in astrocytoma patients [overall survival (OS): *p* = 0.0011; progression‐free survival (PFS): *p* = 0.004]. ZM fusion was associated with a worse prognosis in both astrocytoma (OS *p* < 0.001, PFS *p* < 0.001) and glioblastoma (OS, *p* = 0.252; PFS, *p* = 0.010) patients. We highlight the utmost relevance of ZM fusion as an adverse prognostic factor in astrocytoma (11/382, 2.88%) and glioblastoma grade 4 (11/401, 2.74%) patients and suggest that the grading of these tumors should be refined.

## Introduction

Gliomas are among the most common tumors of the central nervous system, accounting for nearly 80% of malignant brain tumors [1]. Gliomas are notorious for their high malignancy and significant heterogeneity [1, 2]. Diagnoses of glioma with genetic characteristics were further refined in the newest 2021 WHO Classification of Tumors of the CNS classification (WHO CNS5) [3]. The status of genetic alterations also plays a pivotal role in determining the classification and grading of gliomas in addition to their histological features [4, 5, 6].

The proto‐oncogene *MET* encodes the tyrosine kinase receptor of hepatocyte growth factor (HGF), which has pivotal functions in cancer biology [7, 8, 9]. Dysregulation of mesenchymal–epidermal transition factor (MET) and its downstream signaling pathways profoundly influences tumor progression and shapes the immune response, underscoring its multifaceted impact on oncogenesis [10, 11]. *MET* fusions and splicing variants (*MET* F/SVs), including *MET* exon 14 skipping, are associated with *MET* overexpression and hyperactivation of MET signaling pathways [12, 13, 14]. Bao *et al* first identified the ZM fusion gene (*PTPRZ1‐MET* fusion gene) as a common type of *MET* fusion gene in glioma, which was associated with a poor prognosis [15]. ZM fusion induces *MET* overexpression, triggering cell proliferation, angiogenesis, and temozolomide resistance, and promotes glioma progression to the more malignant *IDH‐*wildtype glioblastoma (GBM) [16, 17]. The overexpression of *MET* and ZM fusions activates MET‐STAT3 downstream signaling and leads to tumorigenesis [18]. ZM fusion also activates the STAT3/ISG20 pathway to increase the migratory ability of tumor cells [19]. ZM fusion has been included in the latest WHO CNS5 as a frequent fusion partner of *MET*. Liu *et al* suggested that low‐grade astrocytomas and *IDH* mutants with F/SVs should be designated as grade 4, as they are associated with worse survival [20]. ZM fusion‐targeted therapy with vebreltinib (PLB‐1001) is currently under evaluation in phase III clinical trials [21], providing a promising therapeutic option for *MET* F/SVs.

Considering the updates in the classification and grading criteria, we reclassified and analyzed multicenter data to provide a comprehensive overview of *MET* F/SVs. We analyzed the clinical and sequencing data to evaluate the incidence of *MET* F/SVs and the survival outcome of patients harboring *MET* F/SVs according to the latest WHO CNS5 classification. This study includes the currently largest glioma cohort with *MET* sequencing data, leveraging the revised grading standards to obtain a detailed profile of *MET* F/SVs.

## Materials and methods

### Ethics approval

The research involving experiments on human subjects met the ethical standards of the Helsinki Declaration. Ethical approval was obtained from the Institutional Review Board of Beijing Tiantan Hospital, Capital Medical University (Approval No. IRB KY2013‐017‐01), and all patients/relatives had provided written informed consent.

### Patient selection

A total of 1,098 patients [963 patients from the Chinese Glioma Genome Atlas (CGGA) database; 135 patients from the TruSight Tumor 170 (TST‐170) study] were enrolled in this research. *MET* F/SVs data were collected from these two cohorts [22, 23]. All patients were classified according to the WHO CNS5 grading system. We re‐examined the hematoxylin and eosin (H&E)–stained slides archived in the CGGA database for each case, including whole‐slide scanning and evaluation of the entire formalin‐fixed, paraffin‐embedded (FFPE) tissue sections. Histopathological features, including microvascular proliferation and the presence of necrosis, were systematically assessed. Molecular pathological information (e.g., *CDKN2A/2B*, *TERT*, and *EGFR* alterations) was retrieved from next‐generation sequencing data and integrated to fulfill the diagnostic and grading requirements of the WHO 2021 classification. Fifty‐seven patients who lacked necessary diagnostic information were excluded. Follow‐up overall survival (OS) and progression‐free survival (PFS) data were obtained from the CGGA database.

### Statistical analysis

We applied the chi‐square test for categorical variables and Fisher's exact test for continuous variables via the R (4.4.1) packages. OS for the CGGA database was calculated from the day of surgery to death, regardless of cause [23]. PFS was calculated from the day of surgery to progression, as defined by the RANO criteria [24]. We then applied the Kaplan–Meier method for survival curves, the log‐rank test for group comparisons, and Cox proportional hazards regression for multivariate analysis. A *p* value <0.05 (two‐sided) was considered statistically significant.

### Molecular validation

Sanger sequencing was applied to validate the ZM fusion RNAprep pure Tissue Kit (Tiangen Biotech) using primers: forward: 5′‐CCGTCTGGAAATGCGAATCCTAAA‐3′; reverse: 5′‐CAGGCCCAGTCTTGTACTCAGCAA‐3′ followed by cDNA synthesis (RevertAid RT Kit, Thermo Fisher Scientific, Waltham, MA, USA) according to the manufacturers' instructions. *MET* F/SVs were calculated from RNA sequencing data. We detected other genes and molecular profiles characteristically altered using methods described previously [13].

### Immunohistochemistry

Immunohistochemical staining was performed as previously described. In brief, sample sections were deparaffinized, rehydrated, and boiled in citrate buffer (100 °C, 10 min) for antigen retrieval. Then, the sections were incubated with primary antibodies (anti‐c‐MET, 1:200, Abcam ab227637, Cambridge, UK) overnight at 4 °C. After treatment with ethanol containing 3% hydrogen peroxide for 10 min, followed by washing with PBS, the sections were incubated with secondary antibodies (1:100, ZSGB‐Bio, Beijing, PR China) at 25 °C for 1 h. Subsequently, images were acquired *via* an Axio Imager 2 microscope (Zeiss) after staining with 3,3′‐diaminobenzidine and hematoxylin.

## Results

### Epidemiological characteristics of *MET* F/SVs

**Figure 1:**
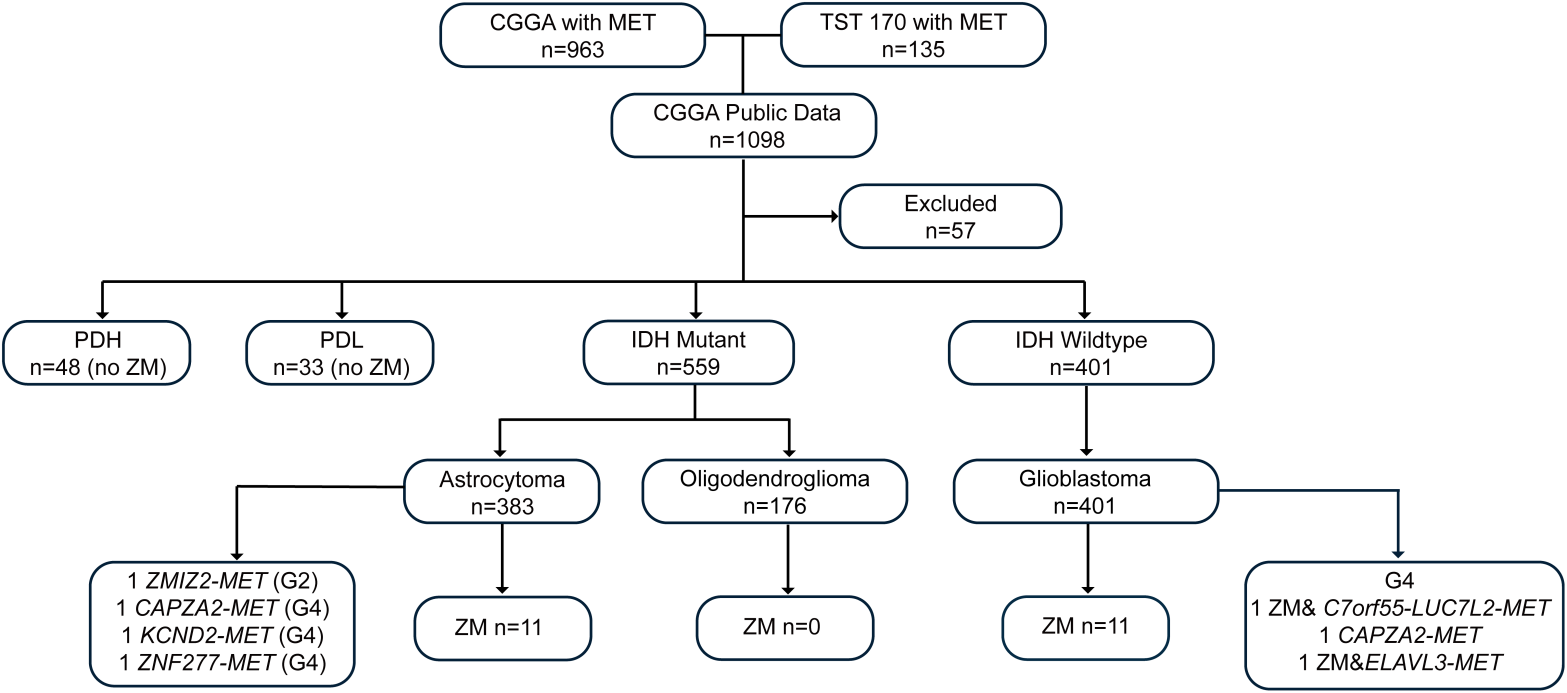
Flowchart of reclassification in the CGGA and TST‐170 databases.

**Figure 2:**
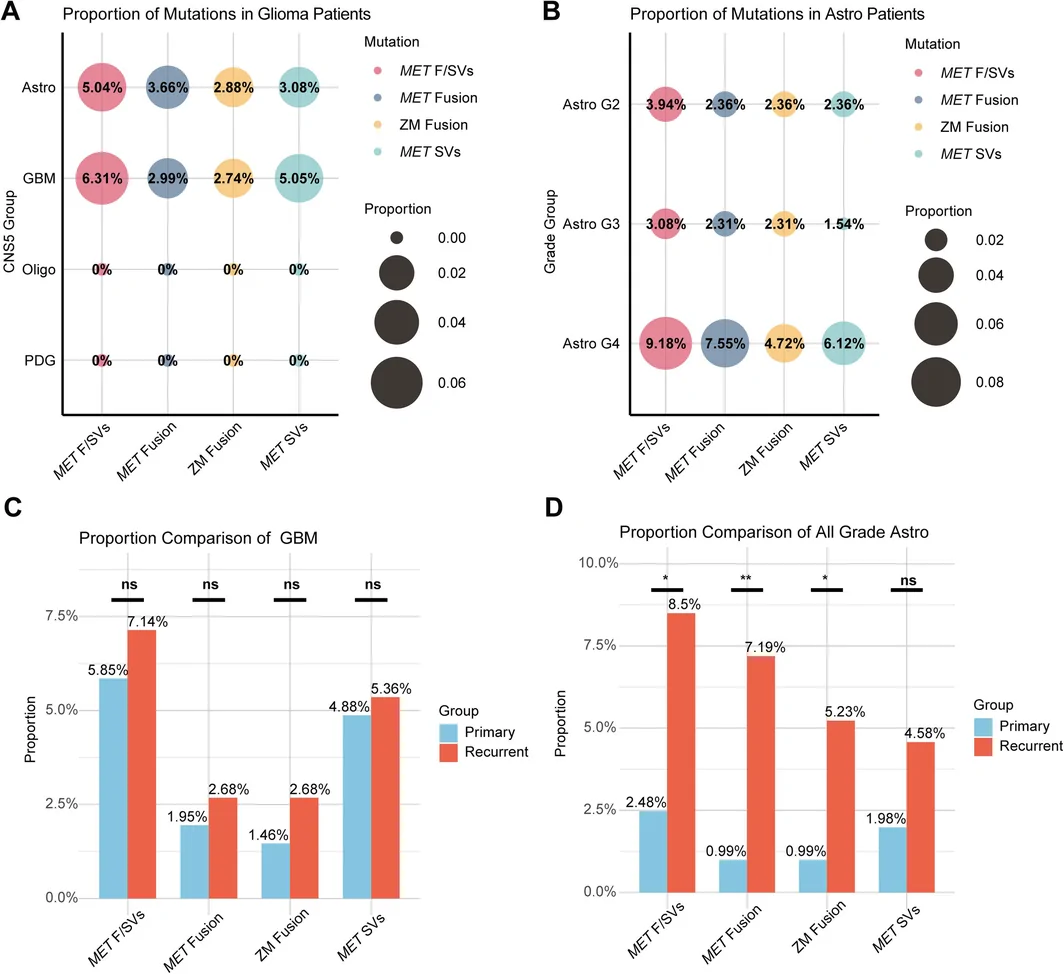
The frequency of *MET* alterations in glioma patients. (A and B) Ratio of different *MET* alterations in glioma patients, stratified by (A) tumor types and (B) astrocytoma grades according to the WHO CNS5 grading system. (C and D) Recurrent patients have a higher ratio of *MET* alteration in (C) GBM and (D) Astro patients. Astro, astrocytoma; CGGA, Chinese Glioma Genome Atlas; G2–G4, grade 2–4; GBM, glioblastoma; Oligo, oligodendroglioma; PDH, pediatric‐type diffuse high‐grade gliomas; PDL, pediatric‐type diffuse low‐grade gliomas. Statistical significance is indicated as follows: *p < 0.05; **p < 0.01; ns, not significant.

A total of 1,041 of 1,098 patients from the CGGA and TST‐170 were finally identified for enrollment and further classified into five subtypes according to the 2021 WHO CNS classification system [22, 23]: astrocytoma, *IDH*‐mutant (Astro, *n* = 383), oligodendroglioma, *IDH*‐mutant and 1p/19q codeleted (Oligo, *n* = 176), GBM (*n* = 401), pediatric‐type diffuse high‐grade gliomas (PDH, *n* = 48), and pediatric‐type diffuse low‐grade glioma (PDL, *n* = 33; Figure 1). The Sankey plot illustrated the transition of patient classifications from the 2016 grading system to the latest system (supplementary material, Figure S1A, B). Among the various *MET* alterations (*n* = 64), fusion accounted for 43.8% (*n* = 28), and SVs represented 56.2% (*n* = 36; supplementary material, Figure S1C). Notably, the occurrence frequency of the ZM fusion gene was significantly greater than that of other *MET* fusion genes (75%, 21/28; supplementary material, Figure S1C), including *ZMIZ2‐MET*, *CAPZA2*‐*MET*, *KCND2‐MET*, *ZNF277‐MET*, *C7orf55‐LUC7L2‐MET*, and *ELAVL3‐MET*. Forty‐nine of the 1,056 patients had F/SVs, and 25 had a ZM fusion (*PTPRZ1‐MET* fusion gene). *MET* F/SVs were detected in only GBM and Astro patients, while none in PDL, PDH, or Oligo patients (Figure 2A). The proportion of *MET* fusions was 3.66% (14/382) in astrocytomas and 2.99% (12/401) in GBMs. ZM fusion alone accounted for 2.88% (11/382) of the cases in Astro and 2.74% (11/401) in GBM.

We further calculated the probability of *MET* F/SVs occurring at different grades within Astro patients. The proportions of *MET* F/SVs (*n* = 9, 9.18%), *MET* fusion (*n* = 8, 7.55%), ZM fusion (*n* = 5, 4.72%), and exon‐skipping (*MET* SVs, *n* = 6, 6.12%; Figure 2B) were greater in astrocytomas grade 4 (AstroG4, *n* = 98) than in astrocytomas grade 2 (Astro G2) and astrocytomas grade 3 (Astro G3).

Notably, patients with recurrent Astro had a higher ratio than did patients with primary tumors for *MET* F/SVs (13/153, 8.5%, *p* = 0.0136, by Fisher's exact test), *MET* fusion (11/153, 7.19%, *p* = 0.0029, by Fisher's exact test), and *ZM* fusion (8/153, 5.23%, *p* = 0.0223, by Fisher's exact test; Figure 2D). Patients with primary Astro grade 4 had no *MET* F/SVs or *ZM* fusion, and all the *MET* F/SVs (*n* = 8) and *ZM* (*n* = 4) cases were recurrent.

There were no significant differences in age, gender, blood type, extent of resection (gross total resection, partial resection or subtotal resection, total tumor resection or subtotal), treatment after surgery (chemotherapy and radiotherapy), or *MGMT* (O6‐methylguanine‐DNA methyltransferase) promoter methylation between patients with or without *MET* F/SVs and *ZM* fusion (Tables 1 and 2).

**Table 1:** Clinical and molecular characteristics of patients with F/SVs positive and F/SVs negative tumors

Characteristic	F/SVs negative, *N* = 922	F/SVs positive, *N* = 41	*p* *
Mean age (range), years	43.09 (12.14)	42.78 (11.78)	>0.9
NA	1	0	
Gender			0.7
Male	539/922 (58%)	25/41 (61%)	
Female	383/922 (42%)	16/41 (39%)	
Blood group			0.3
A	222/841 (26%)	6/35 (17%)	
B	276/841 (33%)	10/35 (29%)	
AB	91/841 (11%)	3/35 (8.6%)	
O	252/841 (30%)	16/35 (46%)	
NA	81	6	
PRS			**0.004**
Primary	587/922 (64%)	17/41 (41%)	
Recurrent	335/922 (36%)	24/41 (59%)	
WHO CNS5 grade			**0.002**
2	262/913 (29%)	5/38 (13%)	
3	246/913 (27%)	4/38 (11%)	
4	403/913 (44%)	29/38 (76%)	
NOS	2/913 (0.2%)	0/38 (0%)	
NA	9	3	
WHO CNS5 type			**0.002**
Astro	339/913 (37%)	18/38 (47%)	
GBM	297/913 (33%)	20/38 (53%)	
Oligo	163/913 (18%)	0/38 (0%)	
PDH	48/913 (5.3%)	0/38 (0%)	
PDL	33/913 (3.6%)	0/38 (0%)	
Oligo NEC	33/913 (3.6%)	0/38 (0%)	
NA	9	3	
Position			0.3
Lt	209/417 (50%)	9/14 (64%)	
Rt	208/417 (50%)	5/14 (36%)	
NA	505	27	
Resection			0.4
Subtotal	273/664 (41%)	14/29 (48%)	
Total	391/664 (59%)	15/29 (52%)	
NA	258	12	
Chemotherapy			0.4
No	238/841 (28%)	13/38 (34%)	
Yes	603/841 (72%)	25/38 (66%)	
NA	81	3	
Radiotherapy			0.8
No	177/848 (21%)	8/36 (22%)	
Yes	671/848 (79%)	28/36 (78%)	
NA	74	5	
*MGMT*p			0.2
Methylated	435/782 (56%)	24/36 (67%)	
Unmethylated	347/782 (44%)	12/36 (33%)	
NA	140	5	

Data are presented as mean (SD) and *n*/*N* (%). Boldface type indicates statistical significance.

F/SVs, *MET* fusions and splicing variants; *MGMT*p, *MGMT* promoter methylation; NA, not applicable; PRS, primary or recurrent status.

* Wilcoxon rank sum test; Pearson's chi‐squared test; Fisher's exact test.

**Table 2:** Clinical and molecular characteristics of patients with ZM positive and ZM negative tumors

Characteristic	ZM negative, *N* = 946	ZM positive, *N* = 17	*p* *
Mean age (range), years	43.10 (12.15)	41.76 (10.26)	0.5
NA	1	0	
Gender			0.3
Male	553/946 (58%)	12/17 (71%)	
Female	394/946 (42%)	5/17 (29%)	
Blood			0.4
A	227/863 (26%)	1/13 (7.7%)	
B	281/863 (33%)	5/13 (38%)	
AB	93/863 (11%)	1/13 (7.7%)	
O	262/863 (30%)	6/13 (46%)	
NA	83	4	
PRS			**0.004**
Primary	599/946 (63%)	5/17 (29%)	
Recurrent	347/946 (37%)	12/17 (71%)	
WHO CNS5 grade			0.5
2	264/935 (28%)	3/16 (19%)	
3	247/935 (26%)	3/16 (19%)	
4	422/935 (45%)	10/16 (63%)	
NOS	2/935 (0.2%)	0/16 (0%)	
NA	11	1	
WHO CNS5 type			0.3
Astro	347/935 (37%)	10/16 (63%)	
GBM	311/935 (33%)	6/16 (38%)	
Oligo	163/935 (17%)	0/16 (0%)	
PDH	48/935 (5.1%)	0/16 (0%)	
PDL	33/935 (3.5%)	0/16 (0%)	
Oligo NEC	33/935 (3.5%)	0/16 (0%)	
NA	11	1	
Extent of resection			0.2
Gross total resection	597/905 (66%)	9/16 (56%)	
Partial resection	95/905 (10%)	4/16 (25%)	
Subtotal resection	213/905 (24%)	3/16 (19%)	
NA	41	1	
Chemotherapy			0.4
No	245/865 (28%)	6/16 (38%)	
Yes	620/865 (72%)	10/16 (63%)	
NA	81	1	
Radiotherapy			0.5
No	181/870 (21%)	4/15 (27%)	
Yes	689/870 (79%)	11/15 (73%)	
NA	76	2	
*MGMT*p			0.4
Methylated	449/803 (56%)	10/15 (67%)	
Unmethylated	354/803 (44%)	5/15 (33%)	
NA	143	2	

Data are presented as mean (SD) and *n*/*N* (%). Boldface type indicates statistical significance.

*MGMT*p, *MGMT* promoter methylation; NA, not applicable; PRS, primary or recurrent status; ZM, *PTPRZ1‐MET* fusion gene.

* Wilcoxon rank sum test; Pearson's chi‐squared test; Fisher's exact test.

### Association of *MET* expression with *MET* F/SVs

**Figure 3:**
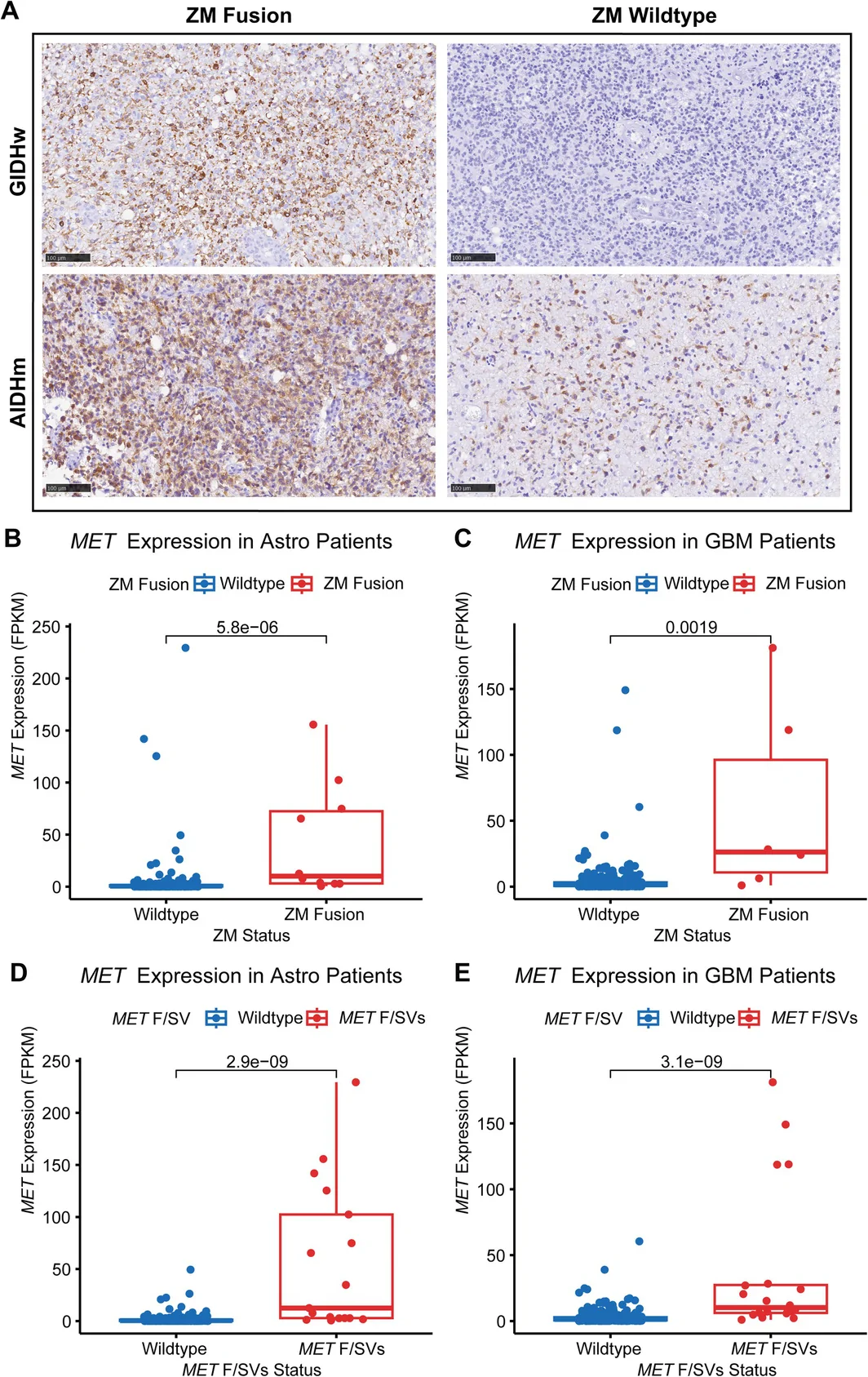
*MET* alterations suggested overexpression of *MET*. (A) IHC for c‐MET in Astro and GBM with (left columns) or without ZM fusion (right columns). Scale bar = 50 μm. (B–E) Box plots of *MET* expression measured as fragments per kilobase of transcript per million mapped reads (FPKM) suggest that (B and C) ZM fusion and (D and E) *MET* F/SVs were associated with higher expression levels of *MET* in (B and D) Astro and (C and E) GBM patients, RNA‐seq data from CGGA. *IDH*m, *IDH* mutant; *IDH*w, *IDH* wildtype.

To characterize the expression profile of *MET* in the *MET* F/SVs group, we applied immunohistochemical assays on glioma tissue for histological comparison between ZM fusion positive and negative glioma. In both GBM *IDH*‐wildtype and Astro *IDH*‐mutant glioma, ZM fusion positive glioma had overexpression of MET (Figure 3A). The elevated *MET* RNA expression was detected in GBM and Astro compared to Oligo (*p* < 0.001; supplementary material, Figure S2A). Compared with Astro G2 (*p* < 0.001) and G3 (*p* = 0.0051; supplementary material, Figure S2B) gliomas, Astro G4 gliomas presented significantly greater *MET* gene expression levels, highlighting a distinct molecular profile associated with advanced‐grade tumors. Boxplots demonstrated that *MET* expression in bulk RNA‐seq showed higher expression levels of *MET* in ZM fusion patients both for Astro (*p* < 0.001; Figure 3B) and GBM (*p* = 0.0019; Figure 3C). The trend of *MET* RNA overexpression was consistent in *MET* F/SVs (Figure 3D, E). We performed UMAP dimensionality reduction on the bulk RNA‐seq data, revealing that patients with the ZM fusion exhibited RNA expression profiles more similar to those of GBM and high‐grade astrocytoma patients (supplementary material, Figure S2C, D).

### Association of *MET* expression with clinical outcomes

**Figure 4:**
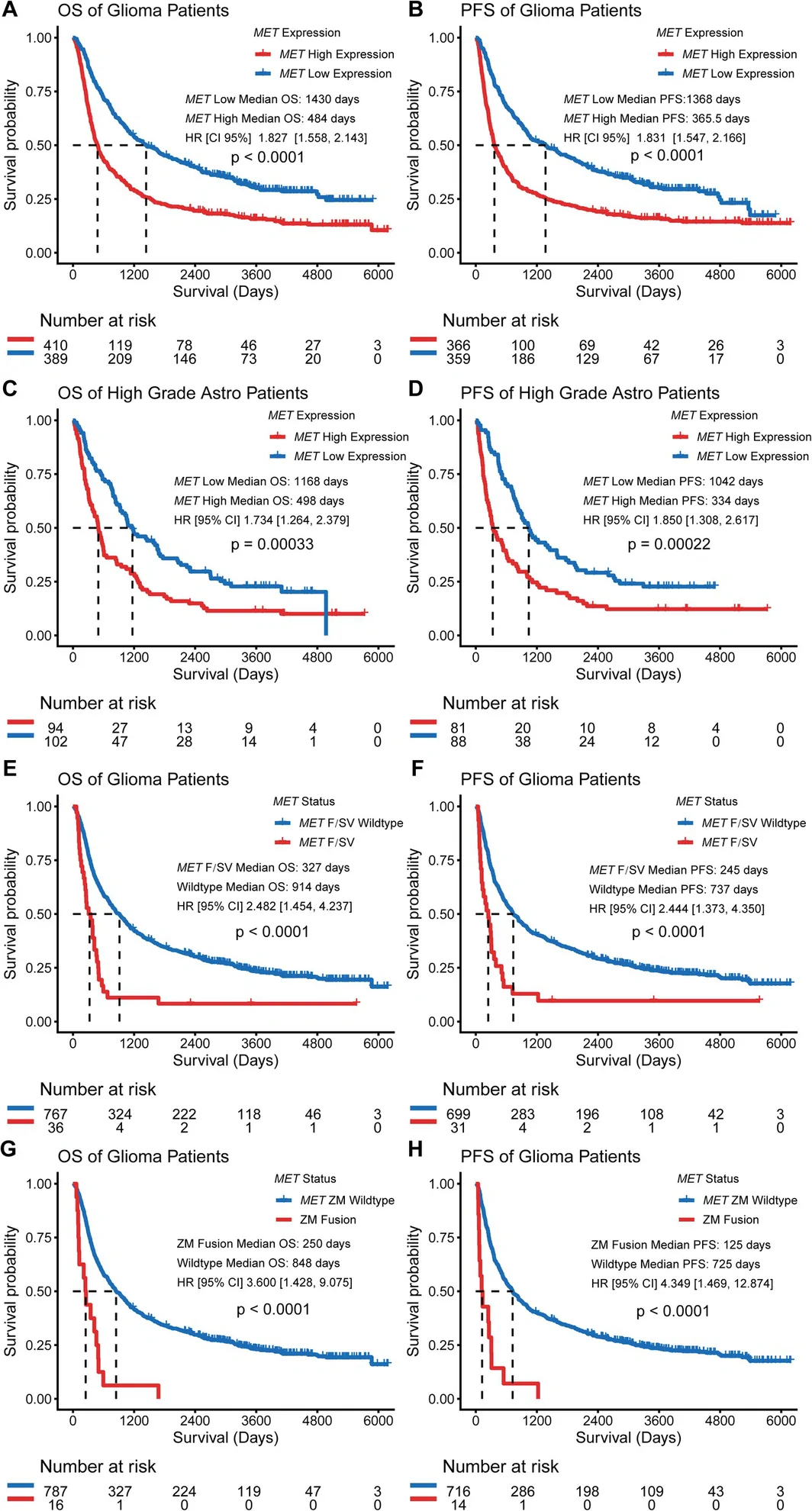
Kaplan–Meier survival analysis in conjunction with the two‐sided log‐rank test showed the association of *MET* high RNA expression, *MET* F/SVs, and ZM fusion with patient prognosis. (A and B) Elevated *MET* RNA expression was associated with worse (A) OS and (B) PFS in glioma patients. (C and D) Elevated *MET* RNA expression was likewise correlated with poorer OS and PFS in high‐grade astrocytoma (grades 3 and 4) patients. (E and F) *MET* F/SVs were significantly associated with worse OS and PFS across glioma patients. (G and H) ZM fusion is linked to worse OS and PFS in glioma patients. OS, overall survival; PFS, progression‐free survival.

Kaplan–Meier (K‐M) survival analysis revealed that patients with *MET* RNA overexpression had significantly shorter OS and PFS (OS: 484 versus 1,430 days, HR 1.827, 95% CI 1.558–2.143, *p* < 0.001; PFS: 365.5 versus 1,368 days, HR 1.831, 95% CI 1.547–2.166, *p* < 0.001; Figure 4A, B). Patients with *MET* RNA overexpression in high grade Astro exhibited poorer prognoses (OS: 498.5 versus 1,168 days, HR 1.734, 95% CI 1.264–2.379, *p* < 0.001; PFS: 334 versus 1,042 days, 1.850 [1.308, 2.617], *p* < 0.001; Figure 4C, D), a trend that was not significant in lower‐grade astrocytomas (supplementary material, Figure S2E, F) or GBM (supplementary material, Figure S2G, H).

Univariate Cox regression analysis indicated that *MET* F/SVs were significantly associated with an increased risk of death and disease progression, corresponding to worse OS and PFS (OS: HR 2.46, 95% CI 1.73–3.51, *p* < 0.001; PFS: HR 2.45, 95% CI 1.67–3.59, *p* < 0.001). ZM fusion was also associated with significantly worse OS and PFS (OS: HR 3.56, 95% CI 2.16–5.88, *p* < 0.001; PFS: HR 4.39, 95% CI 2.57–7.49, *p* < 0.001; supplementary material, Table S1).

K‐M survival curves revealed that patients with *MET* F/SVs had significantly shorter median OS (327 versus 914 days, HR 2.482, 95% CI 1.454–4.237, *p* < 0.001) and PFS (245 versus 737 days, HR 2.444, 95% CI 1.373–4.350, *p* < 0.001) than patients without F/SVs (Figure 4E, F). Patients with ZM fusion also had significantly shorter median OS (250.5 versus 848 days, HR 3.600, 95% CI 1.428–9.075, *p* < 0.001; Figure 4G) and PFS (125 versus 725 days, HR 4.349, 95% CI 1.469–12.874, *p* < 0.001; Figure 4H) than patients without ZM fusion.

### Prognostic relevance of *MET* F/SVs among Astro and GBM patients

**Figure 5:**
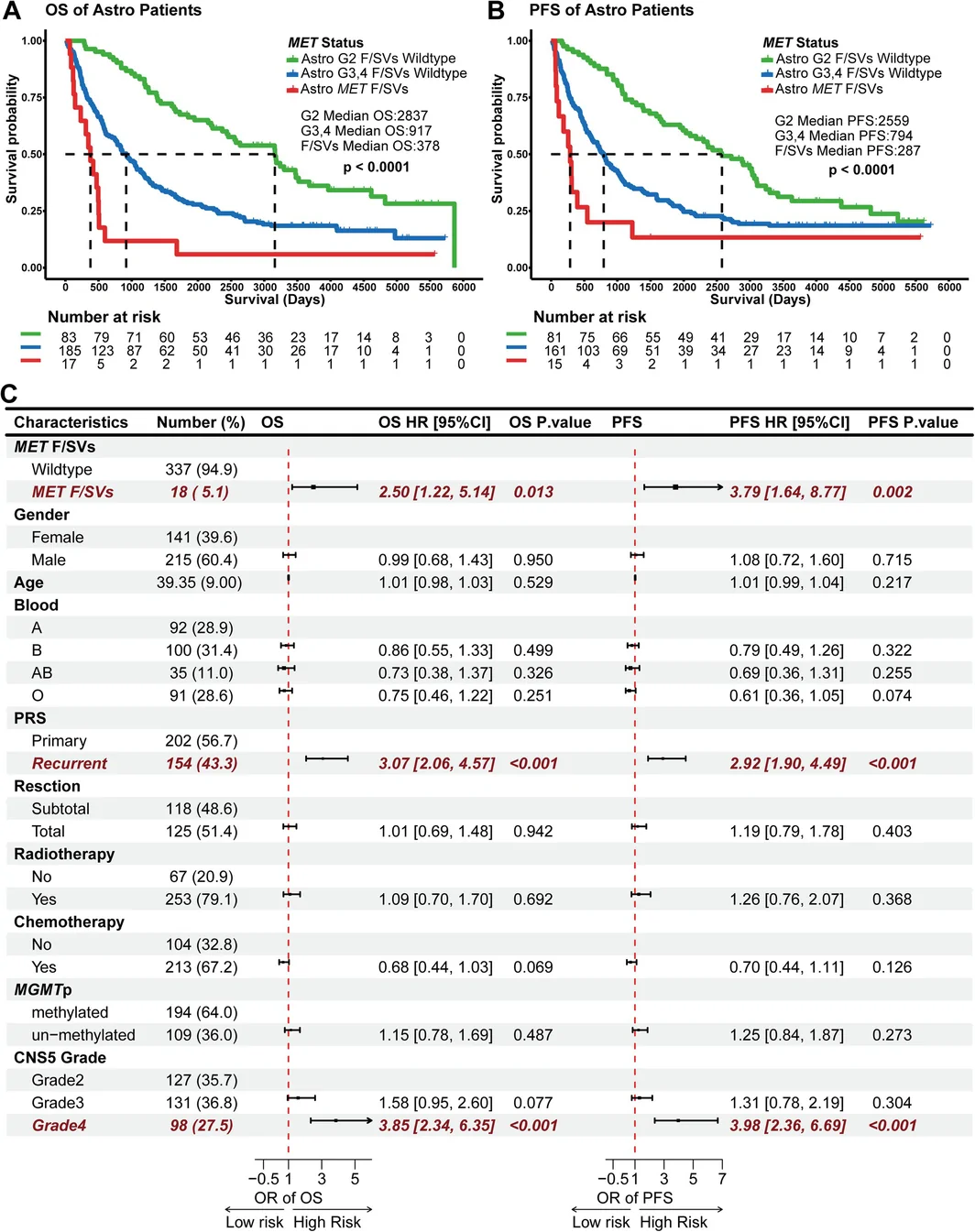
Astro patients with *MET* F/SVs had poorer outcomes. (A and B) K‐M survival curves of (A) OS and (B) PFS in patients of Astro grade 2 (G2), Astro G3/4, and Astro patients harboring *MET* F/SVs. (C) Forest plots showing risk factors for OS and PFS based on multivariate analyses.

In Astro patients, *MET* F/SVs was associated with shorter median OS (378 days; *p* < 0.001; Figure 5A) and PFS (287 days; *p* < 0.001; Figure 5B) comparing to higher grade patients (grade 3 and 4; OS: 917; PFS: 794 days) and low grade (grade 2; OS: 2837; PFS: 2559 days). In multivariate analysis, *MET* F/SVs was independently associated with worse OS (OS: HR 2.41, 95% CI 1.23–4.72, *p* = 0.0011) and PFS (PFS: HR 3.02, 95% CI 1.42–6.45, *p* = 0.004; Figure 5C). Irrespective of tumor grade (multivariate result, OS: HR 5.33, 95% CI 3.39–8.39, *p* < 0.001; PFS: HR 3.55, 95% CI 3.55–9.00, *p* < 0.001) and recurrence status (multivariate result, OS: HR 2.91, 95% CI 2.07–4.08, *p* < 0.001; PFS: HR 2.65, 95% CI 1.87–3.75, *p* < 0.001; Figure 5C), *MET* F/SVs indicated a worse prognosis.

In GBM patients, *MET* F/SVs patients had a shorter median OS [261 versus 357 (GBM grade 4) days, *p* = 0.54; supplementary material, Figure S3A] and PFS (183 versus 279.5 days, *p* = 0.65; supplementary material, Figure S3B). But we did not observe a prognostic relevance of the *MET* F/SVs on OS (HR 1.39, 95% CI 0.80–2.42, *p* = 0.239) and PFS (HR 1.45, 95% CI 0.80–2.64, *p* = 0.222; supplementary material, Figure S3C).

### Prognostic relevance of ZM fusion and *MET* SVs among Astro and GBM patients

**Figure 6:**
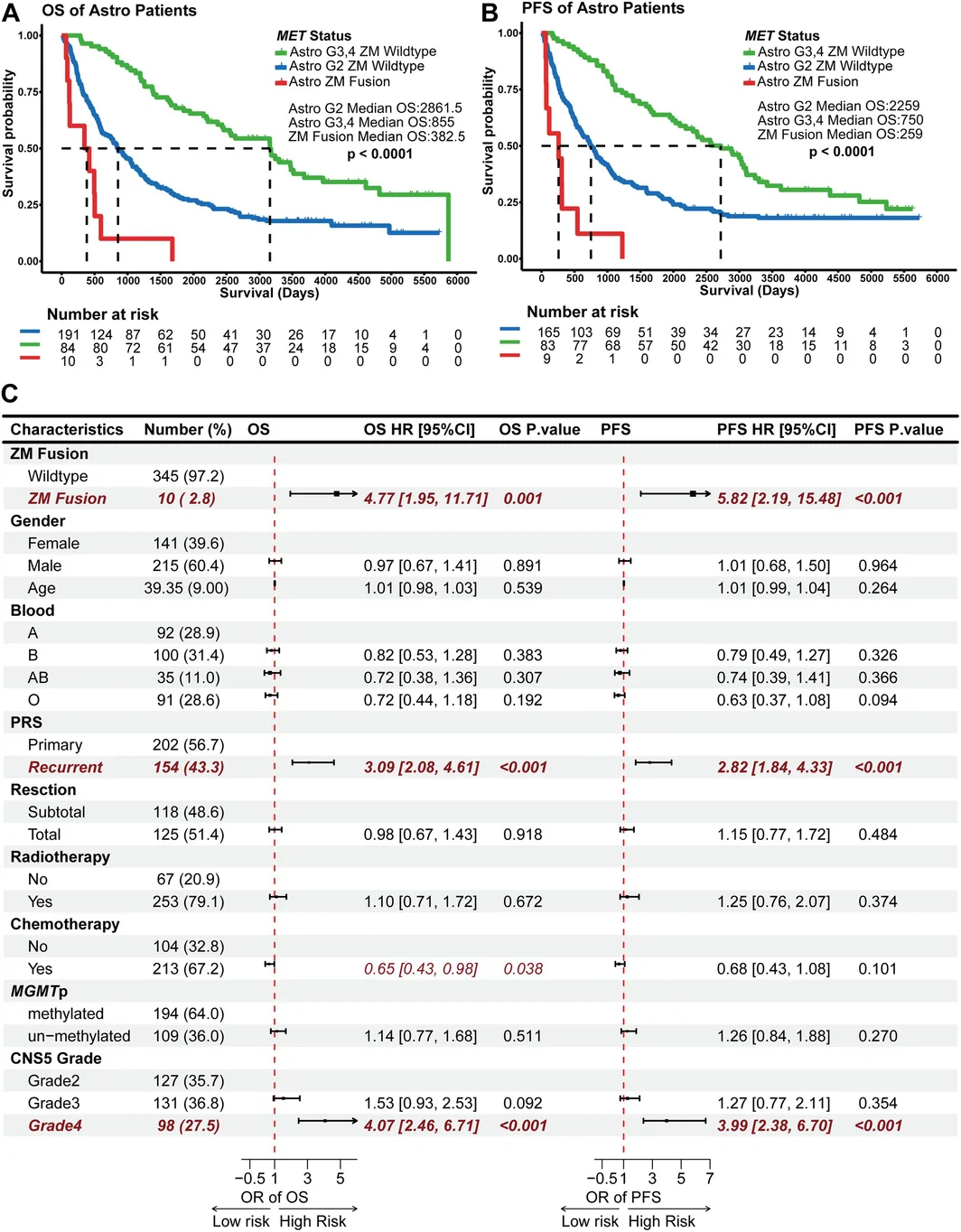
Astro patients with ZM fusion had poorer outcomes. (A and B) K‐M survival curves of (A) OS and (B) PFS in patients of Astro grade 2 (G2), Astro G3/4, and Astro patients harboring ZM fusion. (C) Forest plot suggests ZM fusion was associated with worse prognosis.

Among the *IDH*‐mutant without 1p/19q codeletion (astrocytoma) patients, K‐M survival curves showed that patients with ZM had significantly shorter median OS [382.5 (ZM fusion) versus 855 (high grade) versus 2,861.5 (low grade) days, *p* < 0.001; Figure 6A] and PFS (259 versus 750 versus 2,559 days, *p* < 0.001; Figure 6B). Multivariate analysis showed that ZM fusion was associated with worse prognosis in addition to established prognostic factors such as age and tumor primary or recurrent tumor for both OS (HR 5.14, 95% CI 2.29–11.50, *p* < 0.001) and PFS (HR 5.22, 95% CI 2.20–12.37, *p* < 0.001; Figure 6C).

In GBM patients, ZM fusion carriers have shorter median OS [261 versus 364 (GBM grade 4) days, *p* = 0.016; supplementary material, Figure S4A] and PFS (70.5 versus 279.5 days, *p* < 0.001; supplementary material, Figure S4B). Multivariate analysis showed that ZM fusion was predictive of shorter OS (HR 1.83, 95% CI 0.65–5.11, *p* = 0.252) and PFS (HR 4.98, 95% CI 1.47–16.84, *p* = 0.010; supplementary material, Figure S4C).

Beyond ZM fusion within *MET* F/SVs, we also conducted an analysis on *MET* SVs (splicing variants) to evaluate its prognostic impact. The K‐M survival curves revealed that *MET* SVs were associated with relatively poorer OS and PFS in high‐grade astrocytomas (supplementary material, Figure S5C, D) and GBM (supplementary material, Figure S5E, F). However, Cox multivariate regression analysis revealed no statistical significance (Astro, *p* = 0.091 for OS, *p* = 0.77 for PFS; GBM, *p* = 0.305 for OS, *p* = 0.237 for PFS).

In summary, ZM fusion demonstrated robust prognostic significance in both astrocytomas and GBM.

## Discussion

This study is the first to provide epidemiological data on *MET* F/SVs and the ZM fusion gene based on the latest classification standards. We calculated the frequency of the *MET* F/SVs based on multicenter data, along with baseline clinical characteristics. Our previous study based on the WHO 2016 CNS grading system suggests a higher ratio of ZM fusion and *MET*ex‐14 in secondary GBM (GBM patients with a history of low‐grade glioma) [13]. Now, under the latest WHO CNS 5, ZM fusion‐positive secondary GBM can be reclassified into grade 4 recurrent astrocytoma and grade 4 GBM (supplementary material, Figure S1B). This may explain why *MET* F/SVs appeared mostly in Astro and GBM. In recurrent *IDH*‐mutant Astrocytoma grade 4, the ZM gene exhibits the highest prevalence (5.97%).

We found that *MET* F/SVs and the ZM fusion gene led to worse clinical prognoses for OS and PFS than did astrocytoma grade 4, suggesting that these molecular alterations may identify a biologically more aggressive subgroup and could serve as grade‐refining molecular markers. Furthermore, multivariate analysis revealed that the ZM gene has prognostic value independent of the CNS5 grading and tumor recurrence type. These findings suggest that ZM fusion defines a subset of astrocytic tumors with particularly aggressive clinical behavior. Although further validation is required, our results indicate that the presence of ZM fusion may warrant consideration as a high‐risk molecular feature in future refinements of grading criteria or risk stratification frameworks, potentially informing diagnostic workflows and therapeutic decision‐making. Huang *et al* devised a highly sensitive clinical diagnostic approach for ZM fusion based on reverse transcriptase PCR, which could enable early detection of ZM fusion [25]. However, the clinical adoption of *MET*‐targeted testing remains limited, primarily due to insufficient awareness of *MET* testing and its therapeutic implications in clinical practice. Therefore, we aimed to highlight the strong prognostic value of the *MET* F/SVs within the CNS5 classification system. Astrocytoma patients with *MET* F/SVs or ZM fusion should be more appropriately classified as grade 4. However, *MET* F/SVs do not appear to function as an independent prognostic factor in GBM. We hypothesize that this may be attributed to the limited sample size and the generally poor prognosis of GBM patients, which could dilute the overall prognostic impact of the *MET* F/SVs. Therefore, we plan to expand future cohorts to include more patients with *MET* F/SVs, aiming to strengthen the evidence base. Nevertheless, *MET* F/SVs positive gliomas remain a rare entity, highlighting the need for broader collaborative efforts and additional research to better characterize this disease.

The proto‐oncogene *MET* plays a pivotal role in tumorigenesis and progression as described previously. Clinically, MET‐targeted therapies are categorized into three main classes: anti‐HGF antibodies, anti‐c‐MET antibodies, and small‐molecule tyrosine kinase inhibitors (TKIs) [26]. However, the HGF‐targeting antibody rilotumumab (AMG‐102) was discontinued during phase II clinical trials due to concerns over efficacy and safety [27, 28]. Similarly, the monovalent c‐MET inhibitor Onartuzumab (MetMAb) had suboptimal therapeutic outcomes [29]. Small‐molecule TKIs directly target the tyrosine kinase domain, effectively inhibiting protein phosphorylation and activation. Theoretically, this mechanism confers robust efficacy not only in cases of ligand‐independent c‐MET activation but also in patients without MET overexpression, broadening their therapeutic potential. In a preclinical study involving two complementary MET‐driven pediatric‐type diffuse high‐grade glioma (pHGG) mouse models, the type I MET TKI Capmatinib (INC280) combined with radiotherapy significantly prolonged OS and PFS [30]. These findings highlight its potential as a therapeutic option for MET‐driven pHGG. On the other hand, a phase II/III trial focusing on IDH mutant high‐grade gliomas has demonstrated the efficacy and safety of the highly selective ATP‐competitive small molecule MET inhibitor Vebreltinib [21]. Currently, Vebreltinib has obtained approval, with its indication circumscribed specifically to ZM fusions in glioma. To ensure that more glioma patients with *MET*‐related mutations can benefit, future efforts must focus on advancing clinical trials and deepening our understanding of the mechanisms underlying *MET* F/SVs. We anticipate that, in the near future, more therapeutic options targeting *MET* alterations, including *MET* amplification and exon‐skipping, will become available to offer glioma patients enhanced benefits in OS and PFS.

Our study has certain limitations. First, all the patients included were of Asian descent. Further studies involving a more diverse population are imperative to minimize potential ethnic bias. Second, some patients from the publicly available CGGA database were from earlier years and could not be reclassified into a specific grade; instead, they were categorized as NOS (not otherwise specified) or NEC (not elsewhere classified). Finally, as ZM fusion is relatively rare and studies tracking its prognosis are limited, the data for *MET* F/SVs carriers included in this study were relatively insufficient for analysis, which may introduce some bias, particularly among GBM patients. Therefore, further research focusing on *MET* F/SVs is needed to gain a better understanding of its epidemiological characteristics.

In conclusion, we have updated multicenter data with the latest classification criteria of the 2021 WHO Classification of Tumors of the CNS. This study presents imperative data on the epidemiological features of *MET* F/SVs carriers in glioma patients including occurrence rates, clinical characteristics, risk factors, and patient survival.

## Author contributions statement

Data collection and pathological and molecular analysis: ZF, CZ, PW and XL. Clinical data collection: ZF, LL, GL and JD. Analysis and interpretation of the data: ZF, CZ, PW, QC, DZ and YF. Project administration, validation and supervision: YZ and ZB. All authors were involved in the writing of the manuscript and have read and approved the final version.

## Supporting information


**Figure S1.** Sankey plots showing the transition of patients under the 2016 WHO CNS grading system to WHO CNS5
**Figure S2.** Box‐plots of *MET* expression level in different types of glioma and by grade in Astro
**Figure S3.** Clinical outcomes of GBM patients with *MET* F/SVs
**Figure S4.** GBM patients with ZM fusion had poorer outcomes
**Figure S5.** Kaplan–Meier survival curves of patients based on *MET* SVs status
**Table S1.** Univariate analyses for OS and PFS of glioma patients

## Data Availability

The datasets supporting the conclusions of this study are publicly available in the CGGA repository (http://www.cgga.org.cn), under the accession numbers mRNAseq_693 and mRNAseq_325.
