# Supplementary material for: MET fusions and splicing variants in glioma: a landscape integrating clinical, pathological, and survival features
Source: J Pathol Clin Res. 2026 Mar 30;12(3):e70085. doi: 10.1002/2056-4538.70085 (PMC13581103; doi:10.1002/2056-4538.70085)
Supplement: Supplementary file 1 [file CJP2-12-e70085-s001.pdf]

MET fusions and splicing variants in glioma: a landscape integrating clinical, pathological, and survival features

Z Fang, C Zheng, P Wang *et al*. *J Pathol Clin Res* https://doi.org/10.1002/2056-4538.70085

Supplementary Figures S1–S5

Supplementary Table S1

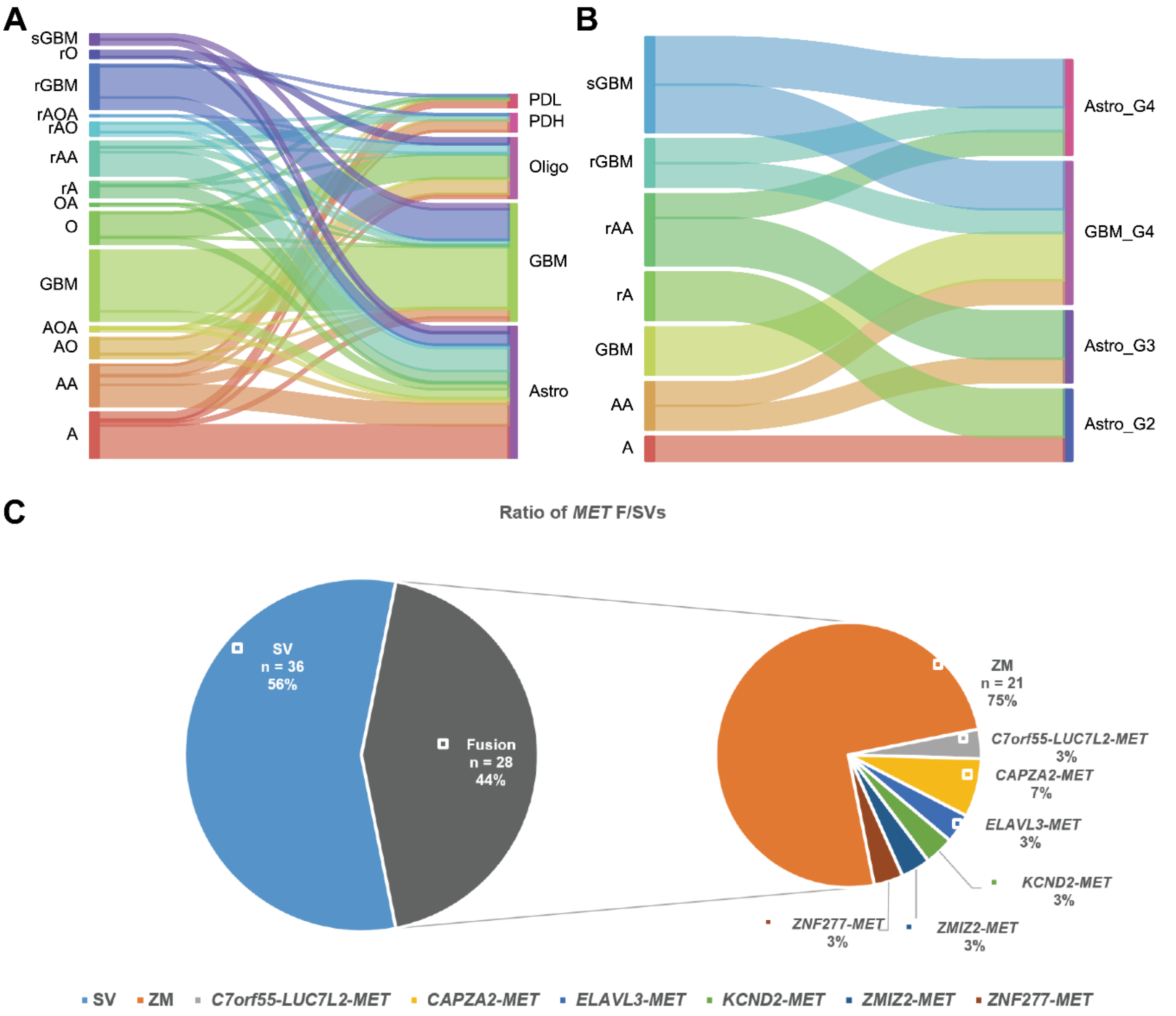

Figure S1. Sankey plots showing the transition of patients under the 2016 WHO CNS grading system to WHO CNS5. (A) Transition of all patients in this study. (B) Transition of patients with ZM fusion. (C) Specific counts of *MET* alteration in *MET* F/SVs patients.

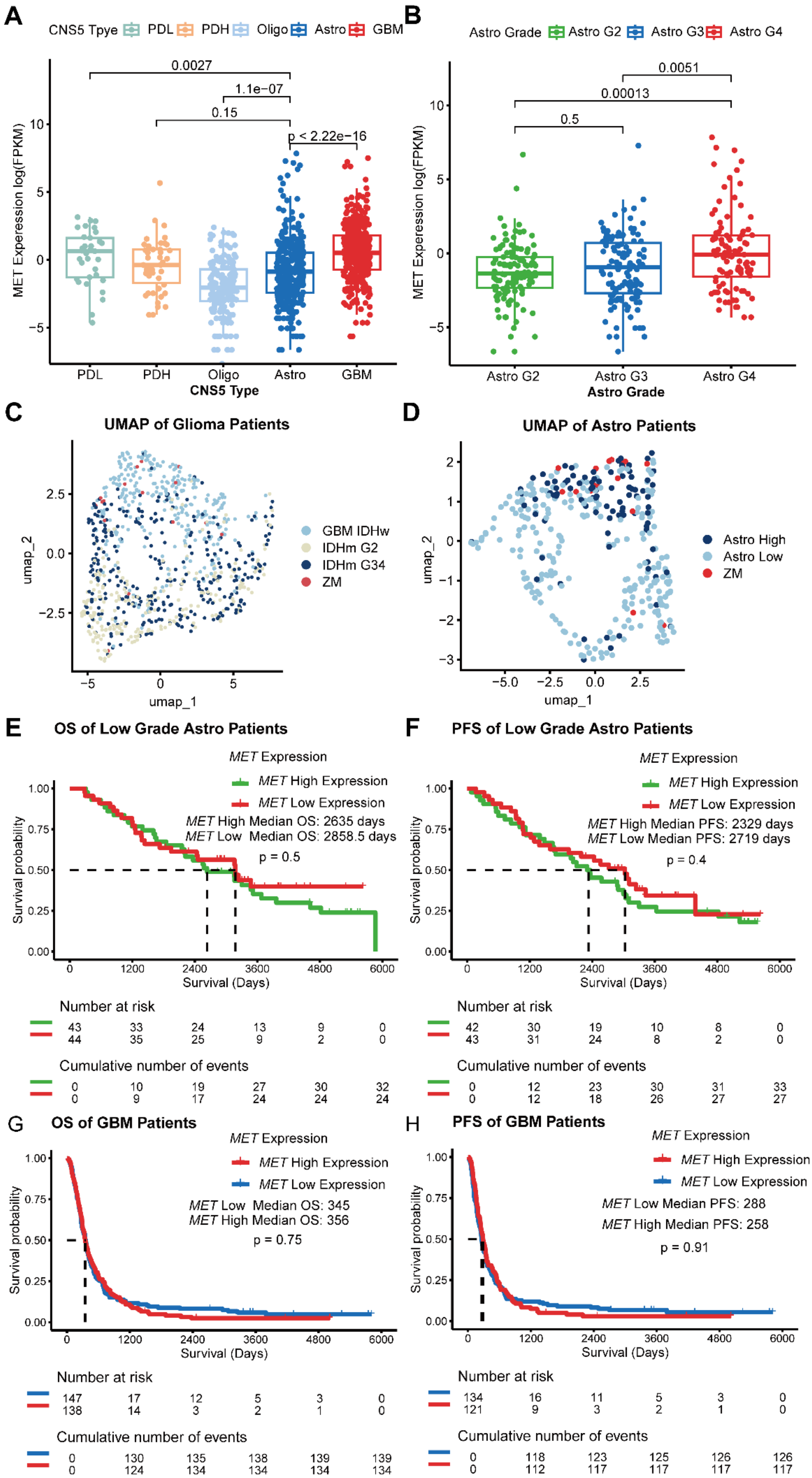
A
CNS5 Tpye PDL PDH Oligo Astro GBM
0.0027
1.1e−07
0.15
p < 2.22e−16
MET Expression log(FPKM)
PDL PDH Oligo Astro GBM
CNS5 Type
B
Astro Grade Astro G2 Astro G3 Astro G4
0.0051
0.00013
0.5
MET Expression log(FPKM)
Astro G2 Astro G3 Astro G4
Astro Grade
C
UMAP of Glioma Patients
GBM IDHw
IDHm G2
IDHm G34
ZM
umap_2
umap_1
D
UMAP of Astro Patients
Astro High
Astro Low
ZM
umap_2
umap_1
E
OS of Low Grade Astro Patients
MET Expression
MET High Expression
MET Low Expression
MET High Median OS: 2635 days
MET Low Median OS: 2858.5 days
p = 0.5
Survival probability
Survival (Days)
Number at risk
43 33 24 13 9 0
44 35 25 9 2 0
Cumulative number of events
0 10 19 27 30 32
0 9 17 24 24 24
F
PFS of Low Grade Astro Patients
MET Expression
MET High Expression
MET Low Expression
MET High Median PFS: 2329 days
MET Low Median PFS: 2719 days
p = 0.4
Survival probability
Survival (Days)
Number at risk
42 30 19 10 8 0
43 31 24 8 2 0
Cumulative number of events
0 12 23 30 31 33
0 12 18 26 27 27
G
OS of GBM Patients
MET Expression
MET High Expression
MET Low Expression
MET Low Median OS: 345
MET High Median OS: 356
p = 0.75
Survival probability
Survival (Days)
Number at risk
147 17 12 5 3 0
138 14 3 2 1 0
Cumulative number of events
0 130 135 138 139 139
0 124 134 134 134 134
H
PFS of GBM Patients
MET Expression
MET High Expression
MET Low Expression
MET Low Median PFS: 288
MET High Median PFS: 258
p = 0.91
Survival probability
Survival (Days)
Number at risk
134 16 11 5 3 0
121 9 3 2 1 0
Cumulative number of events
0 118 123 125 126 126
0 112 117 117 117 117

Figure S2. Box-plots of *MET* expression level in (A) different types of glioma and (B) grade in Astro. RNA-seq data from CGGA. (C and D) UMAP dimensionality reduction of RNA-seq data. (E–H) Kaplan–Meier curves with two-sided log-rank tests illustrating the correlation between MET expression and prognosis in low-grade Astro (E and F; grade 2) and (G and H) GBM patients.

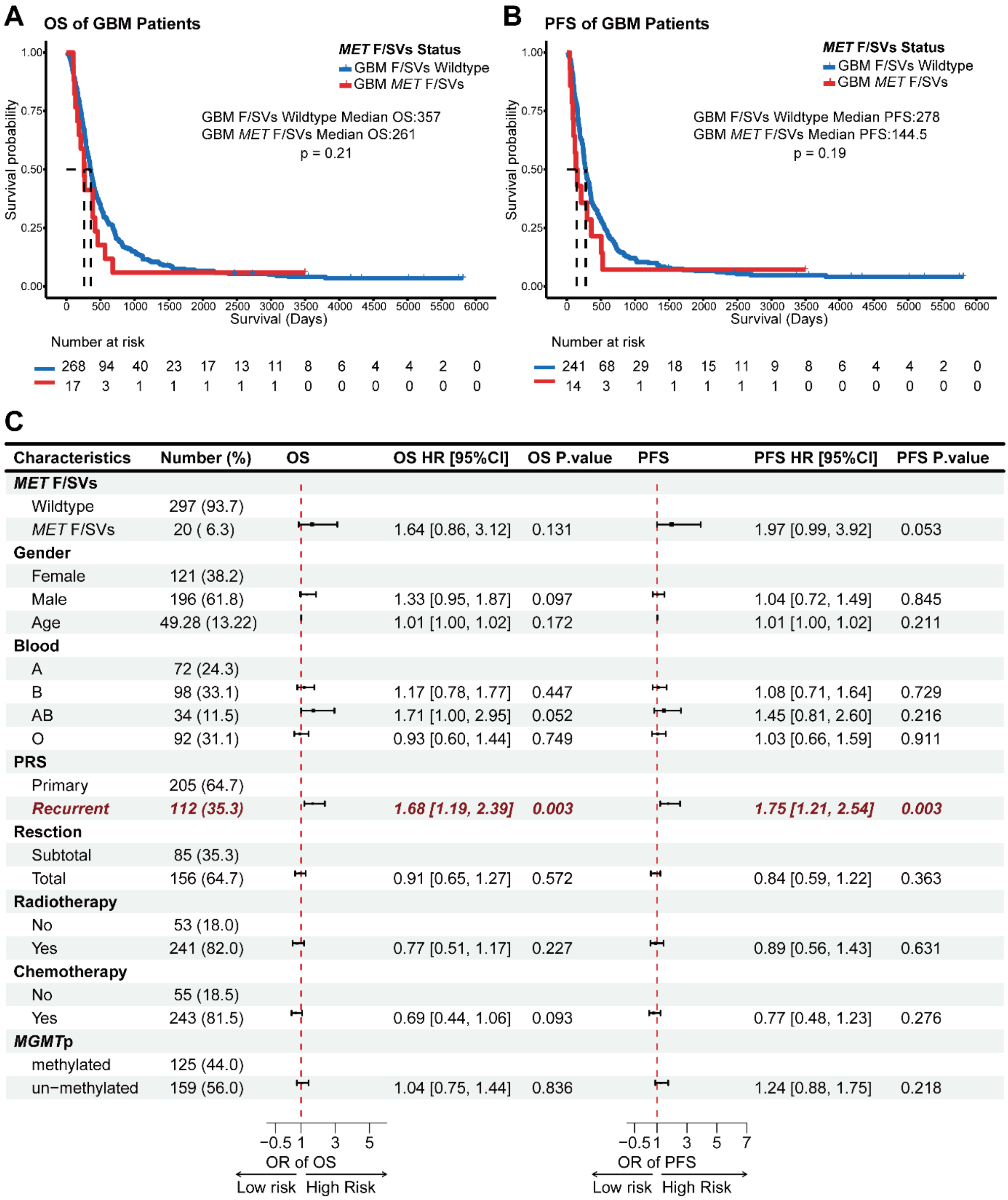

Characteristics	Number (%)	OS	OS HR [95%CI]	OS P.value	PFS	PFS HR [95%CI]	PFS P.value
***MET* F/SVs**							
Wildtype	297 (93.7)						
MET F/SVs	20 (6.3)		1.64 [0.86, 3.12]	0.131		1.97 [0.99, 3.92]	0.053
**Gender**							
Female	121 (38.2)						
Male	196 (61.8)		1.33 [0.95, 1.87]	0.097		1.04 [0.72, 1.49]	0.845
Age	49.28 (13.22)		1.01 [1.00, 1.02]	0.172		1.01 [1.00, 1.02]	0.211
**Blood**							
A	72 (24.3)						
B	98 (33.1)		1.17 [0.78, 1.77]	0.447		1.08 [0.71, 1.64]	0.729
AB	34 (11.5)		1.71 [1.00, 2.95]	0.052		1.45 [0.81, 2.60]	0.216
O	92 (31.1)		0.93 [0.60, 1.44]	0.749		1.03 [0.66, 1.59]	0.911
**PRS**							
Primary	205 (64.7)						
Recurrent	***112 (35.3)***		***1.68 [1.19, 2.39]***	***0.003***		***1.75 [1.21, 2.54]***	***0.003***
**Resction**							
Subtotal	85 (35.3)						
Total	156 (64.7)		0.91 [0.65, 1.27]	0.572		0.84 [0.59, 1.22]	0.363
**Radiotherapy**							
No	53 (18.0)						
Yes	241 (82.0)		0.77 [0.51, 1.17]	0.227		0.89 [0.56, 1.43]	0.631
**Chemotherapy**							
No	55 (18.5)						
Yes	243 (81.5)		0.69 [0.44, 1.06]	0.093		0.77 [0.48, 1.23]	0.276
***MGMT*p**							
methylated	125 (44.0)						
un−methylated	159 (56.0)		1.04 [0.75, 1.44]	0.836		1.24 [0.88, 1.75]	0.218

Figure S3. Clinical outcomes of GBM patients with *MET* F/SVs. (A and B) K-M survival curves showed no difference in GBM patients with or without *MET* F/SVs. (C) Forest plot based on multivariate analyses.

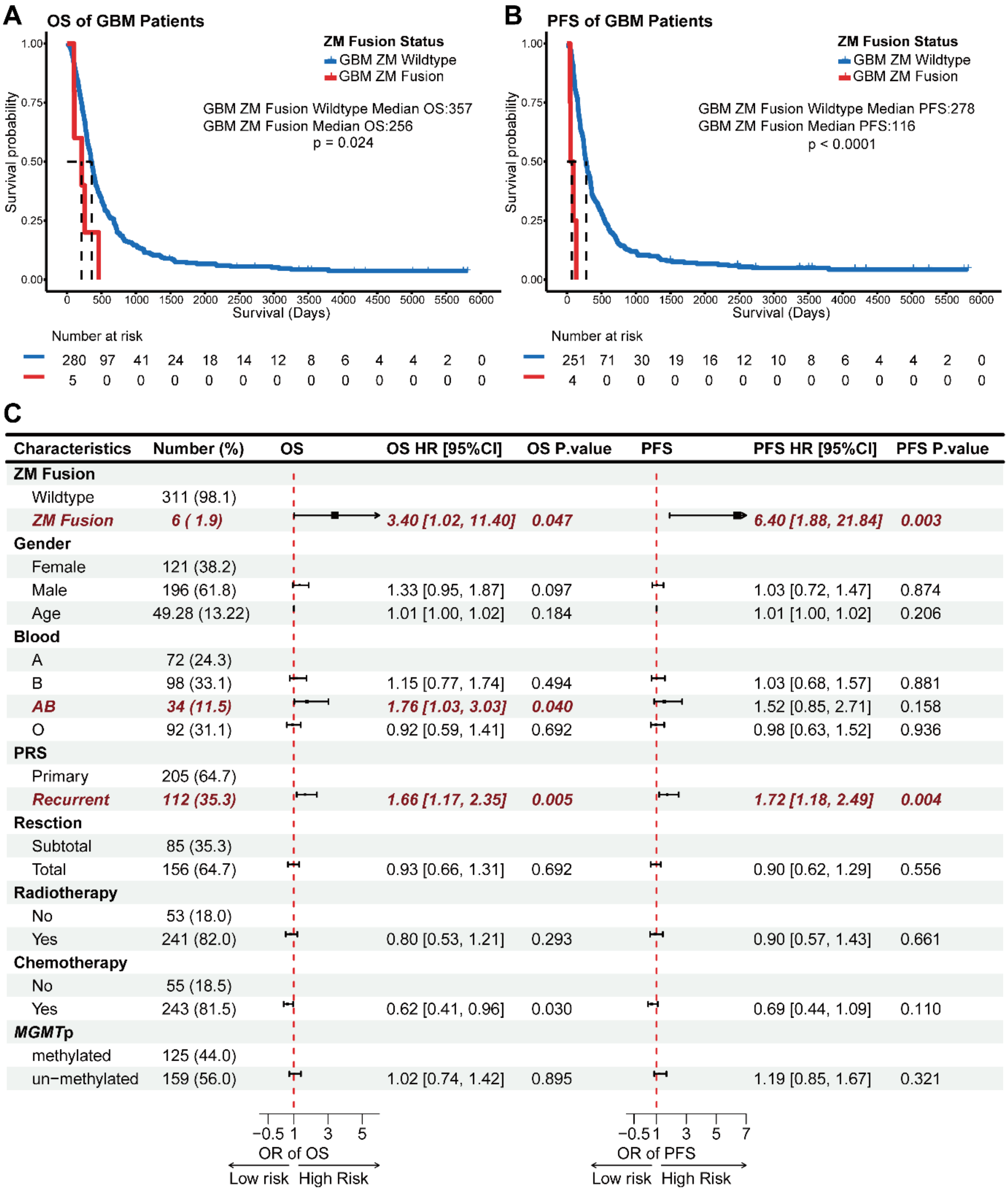

Figure S4. GBM patients with ZM fusion had poorer outcomes. (A and B) K-M survival curves showed that ZM fusion indicating worse OS and PFS in GBM. (C) Forest plot suggested ZM fusion was associated with worse prognosis.

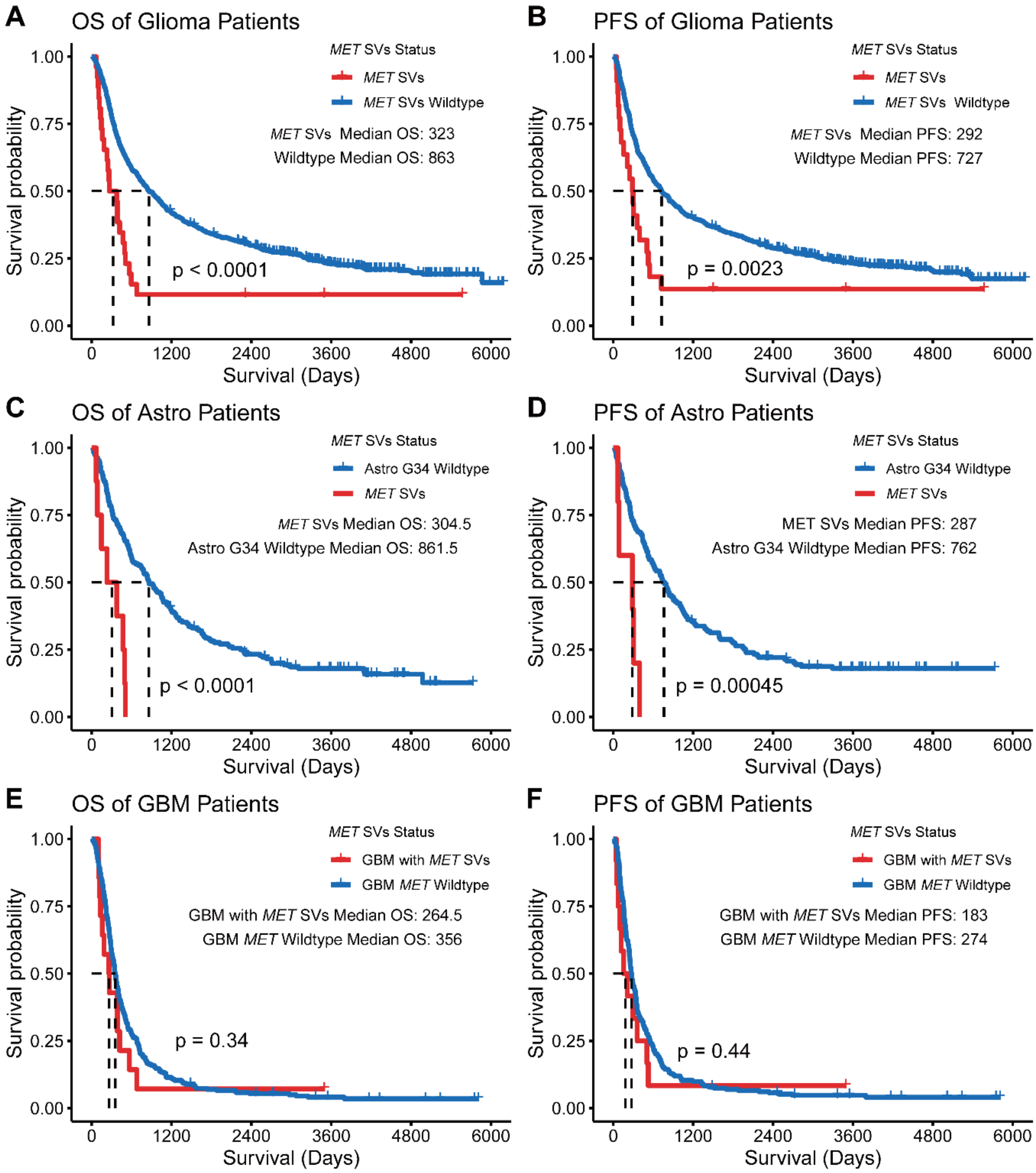

Figure S5. Kaplan–Meier survival curves of patients based on *MET* SVs status. (A and B) *MET* SVs indicating worse (A) OS and (B) PFS in all types of glioma patients. (C–F) K-M survival curves further stratified by glioma subtypes: (C and D) high-grade Astro and (E and F) GBM patients.

Table S1. Univariate analyses for OS and PFS of glioma patients.

Variables[1]	*N* (%)	OS			PFS		
		HR	CI	*p*	HR	CI	*p*
MET F/SVs							
Negative (reference)	878 (95.5)						
Positive	41 (4.5)	2.58	1.81-3.68	**<0.001**	2.49	1.7-3.64	**<0.001**
ZM							
Negative (reference)	902 (98.2)						
Positive	17 (1.8)	4.05	2.45-6.68	**<0.001**	4.96	2.9-8.47	**<0.001**
Age, mean (SD)	42.97 (12.11)	1.03	1.02-1.04	**<0.001**	1.03	1.03-1.04	**<0.001**
Gender							
Female (reference)	382 (41.5)						
Male	538 (58.5)	1.04	0.89-1.23	0.615	1.07	0.9-1.27	0.448
Blood type							
A (reference)	223 (26.6)						
AB	91 (10.9)	0.96	0.71-1.28	0.773	0.86	0.63-1.17	0.335
B	268 (32.0)	0.94	0.76-1.17	0.583	0.87	0.7-1.09	0.235
O	256 (30.5)	0.96	0.77-1.2	0.743	0.97	0.77-1.21	0.773
Extent of resection							
Gross total resection (reference)	576 (65.5)						
Partial	97 (11.0)	1.19	0.91-1.54	0.205	1.09	0.83-1.44	0.546
Gross total versus subtotal	206 (23.4)	0.94	0.77-1.15	0.561	0.88	0.72-1.09	0.242
Radiotherapy							
No (reference)	174 (20.6)						
Yes	669 (79.4)	0.88	0.72-1.08	0.219	0.97	0.78-1.21	0.819
Chemotherapy							
No (reference)	242 (28.8)						
Yes	597 (71.2)	1.28	1.06-1.54	**0.01**	1.39	1.15-1.7	**0.001**
PRS							
Primary (reference)	576 (62.6)						
Recurrent	344 (37.4)	2.43	2.06-2.86	**<0.001**	2.29	1.93-2.72	**<0.001**
WHO CNS5 type							
Astro (reference)	356 (39.2)						
GBM	308 (33.9)	3.39	2.81-4.09	**<0.001**	3.39	2.78-4.12	**<0.001**
Oligo	163 (18.0)	0.33	0.24-0.44	**<0.001**	0.38	0.28-0.5	**<0.001**
PDH	48 (5.3)	2.26	1.6-3.19	**<0.001**	2.49	1.74-3.56	**<0.001**
PDL	33 (3.6)	0.48	0.28-0.82	**0.008**	0.45	0.25-0.81	**0.008**
WHO CNS5 grade							
Grade 2 (reference)	258 (28.4)						
Grade 3	244 (26.9)	2.46	1.9-3.2	**<0.001**	2.22	1.72-2.87	**<0.001**
Grade 4	406 (44.7)	8.04	6.31-10.25	**<0.001**	7.02	5.53-8.9	**<0.001**
MGMTp[2]							
Methylated (reference)	435 (55.7)						
Unmethylated	346 (44.3)	1.23	1.03-1.46	**0.019**	1.34	1.12-1.6	**0.001**

[1]Using Cox proportional hazards model.

[2]*MGMT*p: MGMT promoter methylation.

Boldface type indicates statistical significance.
